# Awareness and knowledge of glaucoma among visitors of main public hospitals in Damascus, Syria: a cross-sectional study

**DOI:** 10.1186/s12886-022-02766-4

**Published:** 2023-01-10

**Authors:** Jameel Soqia, Jamal Ataya, Rawan Alhalabi, Rawan Alhomsi, Romeo Hamwy, Khaled Mardini, Ammar Hamzeh

**Affiliations:** 1 Faculty of Medicine, Damascus University, Damascus, Syria; 2 Faculty of Medicine, University of Aleppo, Aleppo, Syria; 3 Department of Ophthalmology, Damascus University, Damascus, Syria; 4 Al-Mouwasat University Hospital, Damascus University, Damascus, Syria

**Keywords:** Glaucoma, Ophthalmology, Public Health

## Abstract

**Background:**

The main way to prevent blindness from Glaucoma is by early detection and diagnosis; and to do so the awareness must be raised among people where Glaucoma is defined as an acquired chronic optic neuropathy characterized by optic disk cupping and visual field loss. Lack of knowledge about this disease is one of the most important reasons that made it develop to advanced stages. Based on that, we conducted scientific research to assess peoples’ awareness and knowledge about Glaucoma.

After reviewing the literature, it was found that this study is the first in Syria.

**Methods:**

We conducted a cross-sectional study in May 2022. The study included participants, who are above 20 years old, from visitors of Al-Mouwasat University Hospital in Damascus, Syria. During one week, Data was gathered according to the questionnaire, which was presented through face-to-face interviews with participants. We have allocated one point (1) to each question. Three levels of knowledge were adopted, namely; weak level from (0) to (3), average level from (4) to (7) and good level from (8) to (11). Associations between participants' demographic and other details were tested using Chi-square test and other tests, and a *p*-value of < 0.05 was considered significant.

**Results:**

A total of 500 participants were interviewed. For awareness of Glaucoma, 33.6% of the participants (*n* = 168) had heard of Glaucoma, and 66.4% hadn’t (*n* = 332). Mean test results for the knowledge of Glaucoma was 1.62 out of 11, and only 8% of participants (*n* = 40) had a good knowledge of Glaucoma. Education Level, governorate, and department of hospital that the patient came for significantly affected the knowledge of Glaucoma. Moreover, hospital, Ophthalmologists' Clinics, and health staff (M = 5.45) were the better Resource for information than family, relatives, and friends (M = 3.16). Finally, social media and the Internet group had the lowest mean test results (M = 1.23). These test results were significant, with a *p*-value < 0.001.

**Conclusion:**

The percentage of knowledge and awareness was significantly low. Organized community awareness methods must disseminate more ability to increase the general public's understanding to avoid injury and late diagnosis of Glaucoma.

**Supplementary Information:**

The online version contains supplementary material available at 10.1186/s12886-022-02766-4.

## Introduction

The main way to prevent many diseases is by early detection and diagnosis, also one of the most important of these diseases is Glaucoma; where Glaucoma is defined as an acquired chronic optic neuropathy characterized by optic disk cupping and visual field loss, and it is usually associated with elevated intraocular pressure [1] and this disease is the second blinding eye disease [2] where about 60 million people around the world suffer from glaucoma [1] and it was found that a third percentage of patients have progressed to blindness before they see doctors or get medical care [3], and this is because of the lack of awareness and knowledge about this disease and its symptoms so the patient ignore its early signs and thus fail to apply treatment at the right time [4, 5]. As awareness and knowledge about glaucoma greatly influence the treatment-seeking behavior of individuals. The lack of awareness is widespread in many countries of the world and is not limited to a specific place where previous studies in Africa reported that the level of awareness and knowledge of glaucoma among Nigerians and Ghanaians is low, as well as among Caucasians [6, 7]. The prevalence of glaucoma in India is estimated at 11.9 million and most cases are detected late as well [8].

Additionally, there is lack of studies evaluating awareness of eye diseases in general, especially Glaucoma in the Middle East, where high level of awareness of glaucoma among Jordanians and Saudis but low-to-average knowledge about it [9, 10], so it was necessary to conduct assessment studies of awareness in more countries in the region like Syria, Lebanon and others.

Based on that, we conducted scientific research to assess the awareness and knowledge of people about Glaucoma, through which we hope that the development and progress in the diagnosis and treatment of glaucoma will be achieved through many proposals that we will present depending on the result of this research.

## Method

This study is a hospital-based cross-sectional study. We have studied the extent of awareness about Glaucoma among visitors to the central governmental hospital in Damascus, Syria, "Al-Mouwasat hospital". The interviews were conducted randomly by visiting the hospital departments between 16/5/2022 and 22/5/2022. We selected people randomly in the waiting rooms and hall and then who agreed to be interviewed were asked. Five hundred individuals over the age of 20 were registered by random sampling, where participation in the survey was voluntary. However, patients and companions were allowed to participate.

Reading assistance was provided to illiterate participants, and clarifications, if necessary, to any of them without affecting their response. Furthermore, researcher bias was checked, sometimes by reviewing response trends in terms of age or education level.

The study was approved by the Ethical Committee of the Faculty of Medicine at Damascus University on 15/5/2022 with a serial number (3116) and complied with the principles of the Helsinki Declaration. Informed consent was obtained from all participants before their inclusion in the study.

### Data collection and questionnaire

Full questionnaire was mentioned with its correct answers in Additional file 1.

The questionnaire was adapted from a previous studies [11–13]. It was initially prepared in English, translated into Arabic (the local language) by language experts, and re-translated into English to check consistency in the meaning of words and concepts. A pilot study was conducted on 50 visitors, and the questionnaire was reviewed according to the primary statistical study. Also, the survey was tested for reliability using the Cronbach's Alpha test. Internal stability of (0.789).

Almost all questions are closed. The questionnaire consists of three parts: sociodemographic or background information, questions to measure clinical characteristics and awareness of glaucoma, and sources of information and knowledge of glaucoma. Data were collected through face-to-face interviews by the authors.

We have allocated one point (1) to each question from the second questionnaire group for each true answer and zero points (0) to the wrong answers. Three levels of knowledge were adopted, namely; weak level from (0) to (3) were 385 (77%), average level from (4) to (7) were 75 (15%), and good level from (8) to (11) were 40 (8%).

### Statistical analysis

The data was automatically exported from Google forms to Excel, and analysis were performed using Statistical Package for Social Sciences software package (SPSS Inc., Chicago, IL, USA) version 23. Chi-square, Independent t-test, One-way ANOVA and Pearson correlation tests were used to define the association of variables with the extent of understanding concerning Glaucoma. The sample size (n) was determined by Cochran’s Sample Size Formula with the assumption of 95% confidence level (Z = 1.96), e is the margin of error which is 5%, p is the (estimated) proportion of the population which has the attribute in question which it equals 50% (or 0.5), and q is 1 – p:

$$n=\frac{{Z}^{2}pq}{{e}^{2}}$$

The required Sample size (n) for this study, applying the previous formula, is 385.

## Results

A total number of 500 Participants were interviewed; the mean participants’ age was 44.94. Also, 44% of the sample were males (*n* = 220), and 56% were Females (*n* = 280). Other details and characteristics are shown in Table 1.

**Table 1:** Sample Characteristics

Variables		Frequency	Percent
Sex	Males	220	44.0
	Females	280	56.0
Education Level	Uneducated	89	17.8
	Primary School	261	52.2
	Secondary School	66	13.2
	College (Not Graduated)	21	4.2
	Graduated	37	7.4
	Institution	26	5.2
Governate	Damascus (Capital)	111	22.2
	Rif Dimashq	124	24.8
	Daraa	32	6.4
	AL-Qunaitera	27	5.4
	Al-swidaa	16	3.2
	Homs	19	3.8
	Hama	23	4.6
	Aleppo	34	6.8
	Idlib	12	2.4
	Lattakia	7	1.4
	Tartus	8	1.6
	Der ALzour	50	10
	Al-Hasaka	14	2.8
	AL-Raqqa	23	4.6
Are your parents’ relatives?	Yes	169	33.8
	No	331	66.2
Are you a Glaucoma Patient?	Yes	12	2.4
	No	488	97.6
Do you have Diabetes?	Yes	76	15.2
	No	424	84.8
Do you have Hypertension?	Yes	93	18.6
	No	407	81.4
Do you have Asthma?	Yes	22	4.4
	No	478	95.6

Moreover, for the awareness of Glaucoma, one question was asked, "if you had heard of Glaucoma of known?", 33.6% of the participants (*n* = 168) had heard of Glaucoma, and 66.4% had not heard of Glaucoma (*n* = 332); also, from these 168 participants, 49 of them had a regular ophthalmology scanning last year, 10 of them had measured their eye pressure once in the last year, and 11 of them had measured their eye pressure twice or more in the last year.

Additionally, awareness and knowledge of Glaucoma had a positive relationship, *p*-value < 0.001. Neither having Diabetes, Hypertension or Asthma had a significant deference on the knowledge of Glaucoma, with a *p*-value of 0.764, 0.428 and 0.861, respectively.

**Fig. 1:**
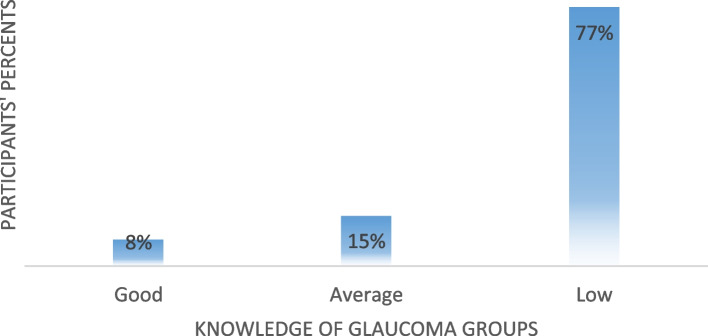
Knowledge of Glaucoma distribution by percent

The mean test results for the knowledge of Glaucoma were 1.62 out of 11. Only 8% of participants (*n* = 40 out of 500) had a good knowledge of Glaucoma (their test results were from 8 to 11), while 15% (*n* = 75) had average knowledge of Glaucoma (test results were from 4 to 7). However, 77% of participants (*n* = 385) had very low knowledge of Glaucoma (Fig. 1). Also, you can see all knowledge questions and their results in Tables 2, 3, 4, 5, and 6.

**Table 2:** Knowledge of Glaucoma Questions and its results

Question	Yes (Right Answer)	No	I don’t know
Risk of Glaucoma increases with age	16%	4.2%	79.8%
Anyone can have Glaucoma?	13.2%	6.4%	80.4%
Blindness from Glaucoma can be prevented	13%	4%	83%
Treatment of Glaucoma is possible	21.8%	1.2%	77%
Glaucoma has Familial Predisposition	11.6%	9.6%	78.8%
Glaucoma has Asymptomatic course	11.2%	10%	78.8%
Is eye pressure different from Arterial Pressure?	19.6%	2.8%	77.6%

**Table 3:** Knowledge of Glaucoma Questions and its results

Question	No (Right Answer)	Yes	I don’t know
Vision is affected in early course	14%	7.2%	78.8%

**Table 4:** Knowledge of Glaucoma Questions and its results

Question	Pressure damage to nerve of vision (Right Answer)	Progressive increase in glasses numbers	Mature Cataract	I don’t know
Results of Glaucoma?	8.2%	2.6%	1.4%	87.8%

**Table 5:** Knowledge of Glaucoma Questions and its results

Question	Slow irreversible loss of vision (Right Answer)	Eyes cannot be operated	Vision is not affected
What will happen in untreated Glaucoma?	12.4%	8.2%	79.4%

**Table 6:** Knowledge of Glaucoma Questions and its results

Question	Only at ocular clinic (Right Answer)	Sphygmomanometer	I don’t know
Measuring Eye pressure by?	20.6%	1%	78.4%

**Fig. 2:**
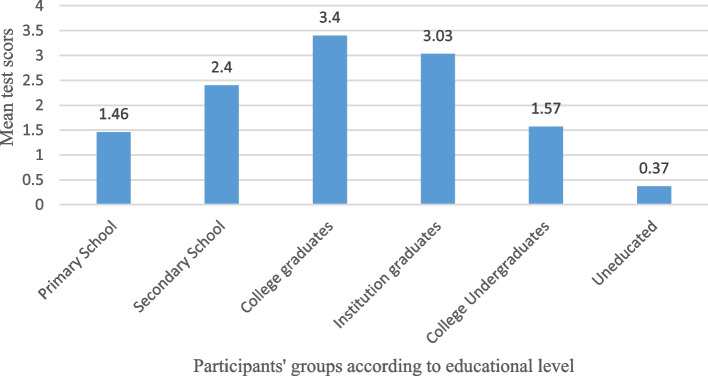
Education Level impact on Knowledge of Glaucoma

A one-way ANOVA was performed to compare the effect of educational level on the knowledge of Glaucoma, there was a statistically significant difference between groups as determined by one-way ANOVA (F(5, 498) = 8.83, *p* < 0.001). A Tukey post hoc test revealed that the knowledge of Glaucoma scores was statistically significantly lower in those who only had a secondary school education (M = 2.04, *p* < 0.001), and primary school education (M = 1.46, *p* < 0.001) and who were undergraduated colleges (M = 1.57, *p* < 0.001) compared to college and institution graduates (M = 3.4, M = 3.03, respectively) (Fig. 2). Also, those who only had a secondary school education and primary school education and who were undergraduated colleges had a significantly higher knowledge than uneducated participants who had the lowest knowledge of Glaucoma with a mean test of 0.37, *p* < 0.001. Each participants’ educational group with their mean Knowledge test results are shown in Fig. 2.

Governorate (the place of living) had a significant difference on the knowledge of Glaucoma, F(13, 498) = 2.43, *p* = 0.003. Rif-Dimashq and Homs participants had a better knowledge of Glaucoma than Aleppo, Der-Alzour and Damascus Participants; test results means were 2.30, 3.36, 0.58, 1.04 and 1.37, respectively. In contrast, neither age nor gender had a significant impact on the knowledge of Glaucoma, with a *p*-value of 0.630 and 0.087, respectively.

There was a significant relationship between the clinic and department of the hospital that the patient came for and knowledge of Glaucoma, *p*-value < 0.001. Which 306 of the participants answered this question, 52 Participants came to the Ophthalmologists’ clinics and had a mean knowledge of 2.38, fifty one participants came to the Cardio clinics and department and had a mean knowledge of 1.21. Also, 18 participants came to Neurosurgery department and had a mean knowledge of 5.05. Other departments and clinics and their participants' mean results are shown in Table 7.

**Table 7:** The Distribution of the Participants among Hospital Departments or clinics

Hospital Departments or Clinics	Number of Participants
Not answered	194
Neurosurgery	18
Neurology	32
Nephrology	26
General Surgery	17
Vascular Surgery	8
Ophthalmology	52
Hematology	26
ENT	24
Pulmonology	13
Cardiology	51
Radiology	4
Orthopedics	12
Urology	5
Thoracic Surgery	1
Internal Medicine	4
Plastic Surgery	1
Endocrinology	4
oncology	1
Rheumatology	1
Gastroenterology	2
Pediatrics	2
Dermatology	2
Total	500

**Fig. 3:**
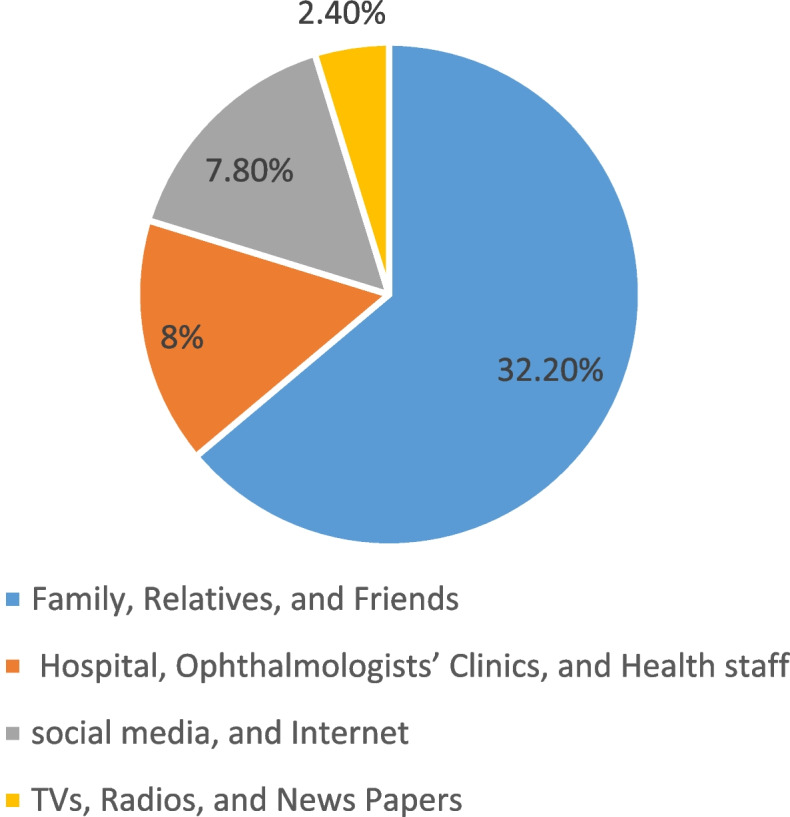
Resource of the Glaucoma information

For the resource of the Glaucoma information, the results were that 2.4% of participants' information came from TVs, radios, and newspapers. In comparison, 32.2% were from family, relatives, and friends. Also, 8% from hospital, Ophthalmologists’ clinics, and health staff. Finally, 7.8% from social media and internet (Fig. 3). Moreover, hospital, Ophthalmologists' clinics, and health staff were the best resource for information (mean knowledge test result = 5.45), then family, relatives, and friends was the second-best result (M = 3.16), while TVs, radios, and newspapers came at third place (M = 2.83). Finally, social media and internet were the worst sources (M = 1.23). These test results were significant, with a *p*-value < 0.001. However, there was no significant relationship between the source of Glaucoma knowledge and the age or the gender of participants.

## Discussion

The aim of this study is to know people's awareness and knowledge of Glaucoma. "Hearing about glaucoma" is defined as "glaucoma awareness". This means that any additional information about Glaucoma is considered "glaucoma knowledge". Despite the prevalence of Glaucoma and it is the second leading cause of blindness worldwide [1], the percentage of awareness about Glaucoma was 33.6%. This is slightly lower than that reported in other studies in Nigeria and Abokobi, which were 36%8, and 39.3%, respectively [5, 14].

Despite the similarity in the percentage of awareness among these countries, we cannot be certain of the similarity for several reasons: the questionnaires are not identical, the method of sample collection, and the place from which the sample was taken. This is about methods, while there are other differences related to the health and awareness system in each of these countries. However, when we talk about the Middle East, in particular, studies from Jordan and Saudi Arabia reported higher rates than what was mentioned in our study [9, 15–17].

So, we will study the factors related and not related to people's awareness, and depending on those factors, we may be able to employ them to spread awareness so that the knowledge rate becomes greater. Among the demographic information, three factors were significantly associated with awareness and knowledge of Glaucoma. The first factor was educational level; this is expected and consistent with previous studies [1, 4, 5, 14, 16, 18–22]. As the more educational levels a person passes, the broader his culture will be to include other fields, including medical. And a part of this medical knowledge may come from personal relationships that include educated people, some of whom may be doctors. And this group of people (educated) may have better access to relevant information from the mass media and other sources than their counterparts [5]. This agrees with a Turkish study which indicates that level of education is the strongest explanatory variable for glaucoma awareness [4].

The second factor is participants with chronic diseases (Diabetes, asthma, Hypertension). These people visit hospitals and clinics from time to time, which means that they are in contact with members of the medical staff. Especially patients with Diabetes and Hypertension. Diabetes is associated with various eye diseases [23], including glaucoma [24], as well as hypertension [25]. Therefore, these patients are supposed to be aware of this complication.

The third factor, the governorate "the place of living", the largest percentage of knowledge was in the Rif Dimashq and Homs. At first, this result can be explained by the fact that Rif Dimashq is a large area and includes many areas. Secondly, social relations in the countryside are closer than in the city, and people are in constant contact. Communication between individuals may contribute to the transmission of medical information about some common diseases such as Glaucoma. But in Homs, awareness campaigns about Glaucoma are organized annually during World Glaucoma Week. Consanguineous marriage is more prevalent in Rif Dimashq and Homs countryside [11]. since Glaucoma is a genetic disorder. It is probably to have a slight increase in the prevalence of Glaucoma in Rif Dimashq and Homs. That makes the residents more aware of Glaucoma.

Our findings show that gender has no relationship with knowledge or awareness, similar to previous studies [1, 4, 5, 9, 18, 20, 22]. There is no relationship between age and awareness of Glaucoma, Like a previous study [9, 15, 18, 20, 26]. And in contrast to previous studies that showed a relationship between older age and awareness [2, 3, 5, 14], another study showed that the knowledge of the youngest was greater, but not by a significant [1].

There was a significant relation between "clinic and department of patients" and "awareness and knowledge of glaucoma". There was higher awareness of Glaucoma in neurosurgery department patients than in ophthalmology clinic patients and cardio clinic patients, respectively. It is probably due to the small sample. Therefore, a sufficient sample should be gained in the case of accurate results. In previous studies, social relations were mentioned as the most important source of knowledge [1, 4, 9, 15, 18, 26, 27], and in other studies, the Internet was the most important [5], health workers [20]. In our study, the most important are social relations. This could include a glaucoma patient and his family, people with a medical culture, or doctors within their families. So, in fact, all these sources are linked to each other. Awareness publishing via the Internet may lead to the creation of knowledge that people will then pass on in their conversations.

Most of the participants thought that glaucoma was not treatable, or at least they didn't know. Despite this, a small percentage of them had their eyes examined for disease. But this can be explained by the lack of knowledge about the asymptomatic course of the disease and that anyone could have it. Among the people who had heard of Glaucoma {168}, {10} had measured their intraocular pressure once, and 11 measured their intraocular pressure more than once. This is a very small and expected percentage, as awareness of Glaucoma alone is not sufficient, but rather needs a good knowledge of the consequences of the Glaucoma, prompting people to get a periodic examination. So it may have a significant relationship with the degree of knowledge and then knowing whether this knowledge was useful or not, but The small number of people who have knowledge about Glaucoma precludes certainty of this relationship.

The low level of awareness and knowledge of Glaucoma among residents of different governorates and ages calls for attention to spread awareness and educate people about Glaucoma and the need for periodic examinations for early detection. There are many means, including:

Publishing medical awareness content on social media platforms.

Educating the glaucoma patients adequately so that they can educate their family and friends well. And prompt them to undergo screening; this may be a good starting point leading to the detection of Glaucoma in the early stages [1]. The education should concentrate on providing the essential information related to the benefits of treatment and early detection, as well as the risk of vision loss [28]. Distribution of pamphlets in hospitals and universities. And organizing awareness campaigns during World Glaucoma Week, which includes an opportunity for free examinations for all patients. It may be especially effective in environments with less admission to higher education and lower literacy rates [22].

The hospital staff also must be aware of the disease in order to improvise case finding and start treatment at an early stage [27].And inform patients of the association of Diabetes and hypertension with glaucoma. And the need for good control of Diabetes and hypertension in addition to the periodic examination of intraocular pressure. By increasing the referral of patients to ophthalmologists, physicians can positively influence screening rates [29, 30].

### Limitation

There are no previous studies conducted in Syria on awareness of glaucoma. In addition, not all the mentioned studies were conducted in hospitals, which affects the accuracy of the comparison. We also need studies that determine the prevalence of glaucoma in Syria.

We did not know if the participants had a family history of glaucoma, in addition, we need a larger sample that includes ophthalmology patients in general, and glaucoma patients in particular, to ensure the extent of their knowledge of their disease. Asking closed-ended questions with multiple options facilitates the statistical process, but some do not have real knowledge about the disease, but the presence of several options prompts them to choose some of them while they are not sure. This type of question creates a fictitious knowledge for the participants. So, we need to make sure the answers of the participants are accurate or not.

## Conclusion

The awareness as much as the knowledge of Glaucoma was shallow. The level of education was the strongest effect factor. The scientific community needs to make more studies to determine variables that control the awareness and knowledge of Glaucoma.

## Supplementary Information

**Additional file 1.**

## Data Availability

The datasets generated and/or analysed during the current study are not publicly available due it is in Arabic language and some restrictions apply to the availability of these data but are available from the corresponding author on reasonable request.
