# Supplementary material for: Awareness and knowledge of glaucoma among visitors of main public hospitals in Damascus, Syria: a cross-sectional study
Source: BMC Ophthalmol. 2023 Jan 10;23:17. doi: 10.1186/s12886-022-02766-4 (PMC9830819; doi:10.1186/s12886-022-02766-4)
Supplement: Supplementary file 1 — Additional file 1. [file 12886_2022_2766_MOESM1_ESM.docx]

The objective of this survey is to know people's awareness and knowledge of glaucoma.

| Answer | Personal information | A |
| --- | --- | --- |
| Male □ Female □ | Gender | A1 |
|  | Age | A2 |
|  | Original governorate | A3 |
|  | Place of living | A4 |
| Uneducated□  Elementary school education□  High school education □  □ Not graduated from university  □Graduated from an institute  □University graduate and above | Level of education | A5 |
| Diabetes □  Arterial hypertension□  Asthma□ | Do you have : | A6 |
| Yes □ No□ | Are your mother and father relatives? | A7 |

| Answer | Awareness question | B |
| --- | --- | --- |
| Yes □ No□ | Have you ever heard of glaucoma? | B1 |

Do you have glaucoma? □ Yes □ No

| Answer | Knowledge questions and Practice | C |
| --- | --- | --- |
| □ Mature Cataract  □ Progressive increase in glasses numbers  □ Pressure damage to nerve of vision  □ I don’t know | Results of glaucoma ? | C1 |
| □ slow irreversible loss of vision  □ eyes cannot be operated  □ I don’t know | What will happen in untreated glaucoma ? | C2 |
| □ Yes □ No  □ do not know | Is eye pressure different from Arterial Pressure? | C3 |
| Sphygmomanometer□  Only at ocular clinic□  do not know I □ | Measuring Eye pressure by ? | C4 |
| □ Yes □ No  □ I do not know | Risk of Glaucoma increases with age? | C5 |
| □ Yes □ No  □ I do not know | Anyone can have glaucoma? | C6 |
| □ Yes □ No  □ I do not know | Vision is affected in early course? | C7 |
| □ Yes □ No  □ I do not know | Glaucoma has Asymptomatic course? | C8 |
| □ Yes □ No  □ I do not know | Treatment of glaucoma is possible? | C9 |
| □ Yes □ No  □ I do not know | Blindness from glaucoma can be prevented? | C10 |
| □ Yes □ No  □ I do not know | Glaucoma has Familial Predisposition? | C11 |
| □ Yes □ No | Have you undergone ocular examination\screening in past one year? | C12 |
| □ Yes, once  □ Yes, more than once  No □ | Have you ever had your Eye pressure Measured ? | C13 |
| □ TV- Radio – Papers  □ Hospitals, Clinics , Clinical Employees  □ Family , Friends  □ Social media | What are your sources of information about Glaucoma? | C14 |
